# Resting‐state connectivity and tobacco smoking in clinical high‐risk for psychosis (NAPLS‐3)

**DOI:** 10.1002/gps3.70002

**Published:** 2026-03-11

**Authors:** Merel Koster, Marieke van der Pluijm, Romy Veelers, Elsmarieke van de Giessen, Lieuwe de Haan, Guido van Wingen, Tim Ziermans, Jentien Vermeulen

**Affiliations:** ^1^ Department of Psychiatry Amsterdam UMC location University of Amsterdam Amsterdam The Netherlands; ^2^ Amsterdam Neuroscience Amsterdam The Netherlands; ^3^ Department of Radiology and Nuclear Medicine Amsterdam UMC location University of Amsterdam Amsterdam The Netherlands; ^4^ Department of Psychology University of Amsterdam Amsterdam The Netherlands

## Abstract

**Background:**

Smoking is highly prevalent among people at clinical high‐risk for psychosis (CHR‐P) and is associated with poorer clinical outcomes. Large‐scale brain networks have been linked to both psychosis and tobacco smoking. However, their relationship in CHR‐P individuals remains unexplored, which may provide valuable insights into the potential neurobiological background of the co‐occurrence.

**Aims:**

The current study aimed to examine whether smoking is associated with altered resting‐state network connectivity over time in CHR‐P individuals.

**Methods:**

Resting‐state functional magnetic resonance imaging scans from the North American Prodrome Longitudinal Study 3 were used. At baseline, 486 CHR‐P non‐smokers and 101 CHR‐P smokers were included, with 1128 scans across 2‐, 4‐, 6‐ and 8‐month follow‐up. Independent component analysis was used to extract functional connectivity for the default mode network (DMN), salience network (SN) and left and right frontoparietal networks (FPN). Differences in within‐ and between‐network strength of the networks of interest were assessed between smoking CHR‐P and non‐smoking CHR‐P at baseline. Linear mixed‐effects models were used to examine associations between longitudinal connectivity changes and smoking.

**Results:**

Results showed that smoking participants were generally light smokers. Smoking was not significantly associated with within‐ or between‐network functional connectivity of the DMN, SN or FPN at baseline or over an 8‐month period in CHR‐P participants.

**Conclusions:**

In this large, longitudinal CHR‐P sample, smoking was not linked to large‐scale functional network connectivity alterations. The early illness stage and limited nicotine exposure may explain the absence of differences, contrasting with findings of reduced network connectivity in schizophrenia and chronic smokers. Future studies could examine connectivity changes over longer periods to determine whether connectivity alterations emerge with increased smoking, illness progression or both.

## Abstract (highlights)

**WHAT IS ALREADY KNOWN ON THIS TOPIC**

- Smoking is pervasive in individuals at clinical high‐risk for psychosis (CHR‐P) and is linked to worse clinical outcomes. Although both smoking and psychosis have been linked to large‐scale brain network alterations, their relationship in CHR‐P individuals remains unexplored.

**WHAT THIS STUDY ADDS**

- Smoking is not associated with large‐scale network changes at this early stage of illness.

**HOW THIS STUDY MIGHT AFFECT RESEARCH, PRACTICE OR POLICY**

- This study provides initial evidence that smoking is not associated with functional connectivity vulnerabilities or changes in large‐scale functional brain networks before the onset of psychosis. However, longitudinal studies with greater smoking exposure and extended follow‐up are needed to determine whether network alterations emerge with heavier use, longer duration or illness progression.

## INTRODUCTION

The alarmingly high smoking rates among individuals with psychotic disorders^1^, ^2^ lead to worse clinical outcomes.^3^ Smokers with a psychotic disorder have higher levels of positive,^4^ negative,^5^ cognitive^6^ and depressive^7^ symptom severity than non‐smoking patients, and research suggests that smoking is implicated in the onset of psychosis.^8^, ^9^, ^10^ Tobacco smoking is linked to a higher‐risk of psychosis,^8^, ^11^, ^12^ with daily use being associated with an earlier onset of psychotic illness.^8^ Additionally, a dose–response relationship between tobacco smoking and the occurrence of schizophrenia has been observed,^9^, ^11^ and one Mendelian randomisation study provided evidence suggesting that smoking may have a causal effect on the development of schizophrenia.^13^

Before the development of a full psychosis, individuals often experience a phase of attenuated, non‐specific symptoms that can last for years.^14^, ^15^ These individuals at clinical high‐risk for psychosis (CHR‐P), who typically exhibit subtle psychotic symptoms or have a family history of psychotic disorders with impaired daily functioning,^16^, ^17^ are particularly vulnerable to developing psychosis within a couple of years.^18^ Notably, the high prevalence of smoking is already present in this prodromal phase of psychosis in CHR‐P individuals.^19^, ^20^, ^21^ This is alarming, as research indicates that ongoing tobacco smoking in CHR‐P individuals is associated with a worsening of psychotic symptoms, whereas reducing cigarette consumption is linked to improvements in affective symptoms.^22^ However, the underlying mechanisms driving the co‐occurrence of psychosis and smoking, or the short‐term biological consequences, remain unclear.

Resting‐state functional magnetic resonance imaging (rs‐fMRI) is a powerful tool for exploring functional brain networks. By examining spontaneous low‐frequency neural activity of the brain, networks of correlated neuronal activity are revealed, offering insights into the intrinsic organisation of the brain.^23^ The increased risk of smoking in psychosis may stem from shared underlying neurobiological vulnerabilities. Changes in neural networks associated with schizophrenia may predispose individuals to nicotine addiction, or conversely, nicotine use may influence the development or expression of symptoms. By examining rs‐fMRI data in CHR‐P individuals with psychosis, we can gain valuable insights into the potential neurobiological background of the smoking–psychosis co‐occurrence. This, in turn, may advance our understanding of the role of smoking in early psychosis and its potential consequences, and support the development of targeted interventions and prevention strategies.

The impact of both psychosis and smoking is complex and widespread throughout the brain, affecting resting‐state functional connectivity in different large‐scale networks.^24^, ^25^, ^26^ Hence, it is important to examine their impact at the network level of the system. The triple network model of major psychopathology identifies the default mode network (DMN), frontoparietal network (FPN) and salience network (SN) as the core neurocognitive networks for understanding cognitive (dys)functioning.^27^ The DMN is typically active during rest and is associated with self‐referential thought; the FPN supports executive functions such as working memory and cognitive control; and the SN detects and filters salient stimuli and facilitates switching between the DMN and FPN.^27^ Mechanistically, nicotine exerts its influence via nicotinic acetylcholine receptors (nAChRs), which modulate neurotransmitter systems, including dopamine, glutamate, γ‐aminobutyric acid and noradrenaline.^28^, ^29^ These systems are involved in regulating brain activity and functional brain networks, including the DMN, SN and FPN.^30^, ^31^, ^32^ Nicotine exposure leads to desensitisation of nAChRs, which could alter resting‐state connectivity by disrupting these systems. Schizophrenia has previously been associated with disintegration within and between these networks.^27^ The DMN, SN and FPN have also proven to be associated with nicotine addiction, and in general, chronic smokers show reduced functional connectivity within the DMN, FPN and SN compared to non‐smokers.^25^, ^33^, ^34^

Regarding CHR‐P individuals, previous studies have demonstrated inconsistent results. One study observed increased functional connectivity between the SN and DMN^35^ compared to controls, and another study demonstrated the absence of DMN‐FPN anticorrelation in CHR‐P.^36^ Furthermore, a recent meta‐analysis examining DMN, FPN and SN network connectivity in CHR‐P individuals found that CHR‐P was associated with reduced connectivity only within the SN. However, this finding did not survive correction for multiple comparisons.^37^ Lastly, one study demonstrated marginal evidence for lower functional connectivity between the SN and DMN in CHR‐P individuals compared to controls.^38^

rs‐fMRI studies evaluating the impact of smoking on the brains of patients with schizophrenia have suggested distinct neural patterns in patients compared to controls.^39^ Regarding large‐scale network connectivity, it has been shown that smoking patients displayed increased connectivity of the DMN to the FPN.^40^ However, no rs‐fMRI studies have investigated the association of smoking and neural network connectivity in CHR‐P individuals. Exploring this relationship may lead to an understanding of potential pre‐existing vulnerabilities in network connectivity that predispose CHR‐P individuals to smoking or the neurobiological consequences of smoking. Therefore, the current study aimed to examine whether smoking is associated with altered resting‐state network connectivity over time in a large number of CHR‐P individuals of the North American Prodrome Longitudinal Study 3 (NAPLS‐3).^41^ Notably, the longitudinal design allowed us to model within‐individual variability, providing a more robust framework for detecting connectivity changes. Specifically, we assessed cross‐sectional differences and longitudinal changes in within‐ and between‐network connectivity in the DMN, SN and FPN between CHR‐P smokers and CHR‐P non‐smokers. Based on previous studies,^25^, ^33^, ^40^ we expected reduced connectivity within the DMN, FPN and SN, and increased connectivity between the DMN and the FPN in smoking compared to non‐smoking CHR‐P individuals. Further, to investigate how CHR‐P participants differ from controls, we also exploratively assessed within‐ and between‐network connectivity changes in CHR‐P participants and controls. Based on earlier studies, we expected increased connectivity between the SN and DMN,^35^ and absence of DMN‐FPN anticorrelation in CHR‐P compared to controls.^35^, ^36^

## MATERIALS AND METHODS

### Participants

Data from the NAPLS‐3 study, conducted from 2015 to 2020 in the United States of America (USA), were used.^42^ Data are publicly available through the National Institute of Mental Health Data Archive. We refer to Addington et al.^41^ for a detailed overview of the study protocols and all procedures.

Participants were aged 12–30 years and were recruited either through referrals from third parties, such as family members or social service agencies, or on their own initiative. Participants were considered CHR‐P when they met the criteria of psychosis‐risk syndromes based on the structured interview for psychosis‐risk syndromes (SIPS).^43^ Attenuated psychotic symptoms were rated on the scale of psychosis‐risk symptoms (SOPS),^43^ and scored as follows: 0 = absent; 1 = questionably present, 2 = mild, 3 = moderate, 4 = moderately severe, 5 = severe but not psychotic and 6 = severe and psychotic. Exclusion criteria for all participants were (1) a history of a psychotic disorder throughout their lifespan, (2) an IQ < 70 and (3) the experience of any neurological disorder. In addition, controls were excluded if they met the criteria for a cluster personality disorder diagnosis, had a first‐degree relative with a history of psychosis or were currently using psychotropic medication. The study procedures were approved by institutional review boards at all participating sites, and informed consent was obtained from all participants or their parents for participants aged < 16 years. Only participants with available data on smoking behaviour and at least one magnetic resonance imaging (MRI) assessment were included in this study.

### Study design and measures

Data were collected in 9 sites across the USA. For both CHR‐P and control participants, demographic characteristics were collected at baseline and neuroimaging and clinical assessments were conducted at baseline and at 2, 4, 6 and 8 months. Clinical assessment included, amongst other things, the SIPS, SOPS and the Global Functioning social and role scales.^44^

Smoking behaviour and cannabis use were evaluated at all time points using the Alcohol and Drug Use Scale.^45^ Tobacco use was rated as follows at each time point: 0 = no use, 1 = occasionally, 2 = < 10 cigarettes per day, 3 = 10–25 cigarettes per day and 4 = > 25 cigarettes per day. Participants were considered smokers if they reported smoking at least occasionally (score ≥ 1). Cannabis and alcohol use were rated as follows: 0 = no use, 1 = once or twice per month, 2 = 3–4 times per month, 3 = 1–2 times per week, 4 = 3–4 times per week and 5 = almost daily in the past month. Subjects were classified as cannabis and/or alcohol users if they reported using it at least once or twice per month (score ≥ 1).

### Image acquisition

High‐resolution whole‐brain T1‐weighted scans were acquired using 3T MRI scanners and a magnetisation‐prepared rapid acquisition gradient‐echo sequence with a standardised set of imaging parameters across all sites.^46^ Additionally, whole‐brain resting‐state functional images were acquired (166 volumes, slice thickness = 3.5 mm, repetition time = 2500 ms, echo time = 29 ms, voxel size = 3.5 mm^3^ isotropic, flip angle = 85°). Participants were instructed to keep their eyes closed and let their minds wander.

### Image preprocessing

Preprocessing was performed using fMRIPrep version 20.12.10.^47^ In short, anatomical scans were normalised to Montreal Neurological Institute space. Motion correction (FLIRT), distortion correction (without fieldmap) and T1w co‐registration were performed as part of the functional data preprocessing. Independent component analysis (ICA)‐based automatic removal of motion artefacts was applied to the fMRI scans. Data were spatially smoothed (6 mm full width at half maximum; Gaussian kernel). Data were further post‐processed by removing the first 2 volumes of the functional data, regressing out average white matter and cerebrospinal fluid signals (obtained after ICA‐AROMA), and applying high‐pass filtering (> 0.01 Hz), all using FSL. Participants were excluded from further analyses if any rotation/translation parameters exceeded 4 mm/degrees, if mean framewise displacement (FD) > 0.3 mm, or if participants had < 80% of motion unaffected data (FD < 0.3 mm).

### Independent component analysis

**FIGURE 1:**
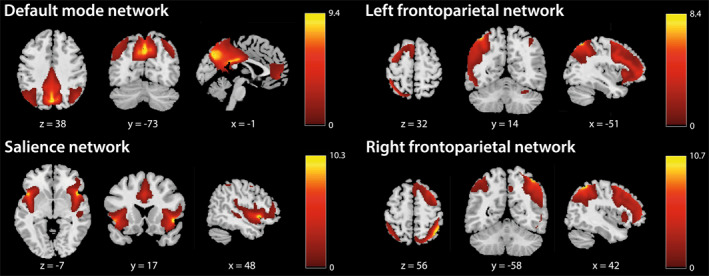
Four canonical resting‐state networks of interest, selected for the group‐level analyses.

We pre‐registered our analysis plan and hypotheses prior to conducting any analyses (https://aspredicted.org/rjqv‐54mq.pdf). Group‐information guided ICA (GIG‐ICA)^48^ was used for the analysis, which decomposes multivariate signals into subject‐specific independent components by simultaneously maximising spatial independence and alignment with predefined spatial network templates at the individual level.^49^ Analysis was performed using the Group ICA Of fMRI Toolbox (GIFT; http://trendscenter.org/software/gift)^50^ in MATLAB (R2022a, Mathworks, Inc.). For the spatial references, spatial maps of 12 canonical resting‐state networks from an independent sample were used^51^ (figure S1). These 12 spatial components were extracted for every subject and session. The resulting maps, containing *z*‐scored weights reflecting the degree to which each voxel's time course contributes to the network of interest, were used for subsequent analysis. The four networks of interest were the DMN, SN and the left and right FPN (figure 1).

### Within‐network strength

To test for changes in within‐network strength, we masked the independent canonical network maps at a *z*‐score of 1.0, based on visual inspection to ensure the networks were neither excessively large nor overly restricted. Next, to obtain a measure of overall within‐network connectivity strength, we computed the mean *z*‐scores of the voxels within the network mask of the subject‐specific component maps for each subject and session using fslmeants.

### Between‐network functional connectivity

Functional network connectivity analyses between the four canonical networks at the subject/session level were performed using the GIFT toolbox.^50^ In short, the between‐network connectivity was obtained by computing Pearson correlations between the time courses of different networks, producing a matrix where each element represents the connectivity between a network pair.^49^ Next, we computed the mean between‐network connectivity for each subject/session across the six combinations of the networks of interest (i.e., DMN × LFPN, DMN × RFPN, DMN × SN, LFPN × RFPN, LFPN × SN and RFPN × SN).

### Statistical analyses

R version 4.3.2^52^ in RStudio (version 2023.12.1, RStudio Inc.) was used for all statistical analyses. If network strength data were not normally distributed, a Yeo‐Johnson transformation was applied for statistical analysis to meet the assumptions of parametric analyses, as it can handle both positive and negative values.^53^ Differences were considered statistically significant at *p* < 0.05, and covariates were included in all statistical analyses: age, sex, cannabis use, alcohol use and MRI site. Alcohol use was additionally included in the pre‐registered covariates, including age, sex and cannabis use, because of the observed significant group differences (table 1).

**TABLE 1:** Demographic and clinical characteristics of CHR‐P participants

	CHR‐P (***n*** **=** **486)**		Statistic	*p*
	Non‐smokers (*n* = 385)	Smokers (*n* = 101)		
Age (years)^a^	18.2 (4.01)	20.2 (3.41)	***t* = −5.17**	< 0.001*^b^
Sex (female), *n* (%)	186 (48)	30 (30)	***χ*** ^**2**^ **= 10.5**	0.001*^c^
Years of education^a^ ^,^ ^d^	11.5 (3.14)	12.9 (2.34)	***t* = −4.95**	< 0.001*^b^
Smoking frequency, *n* (%)			***χ*** ^**2**^ **= 486.0**	< 0.001*^c^
No use	385 (100)	0 (0)		
Occasionally	0 (0)	59 (58)		
< 10 cigarettes per day	0 (0)	22 (22)		
10–25 cigarettes per day	0 (0)	18 (18)		
> 25 cigarettes per day	0 (0)	2 (2)		
Cannabis users, *n* (%)	66 (17)	62 (61)	***χ*** ^**2**^ **= 78.5**	< 0.001*^c^
Cannabis severity, *n* (%)			***χ*** ^**2**^ **= 91.2**	< 0.001*^c^
No use	319 (83)	39 (39)		
1–2 per month	37 (10)	21 (21)		
3–4 per month	7 (2)	9 (9)		
1–2 per week	8 (2)	14 (14)		
3–4 per week	9 (2)	10 (10)		
Almost daily	5 (1)	8 (8)		
Alcohol users, *n* (%)	117 (30)	77 (76)	***χ*** ^**2**^ **= 68.2**	< 0.001*^c^
Alcohol severity, *n* (%)			***χ*** ^**2**^ **= 84.2**	< 0.001*^c^
No use	268 (70)	24 (24)		
1–2 per month	54 (14)	23 (23)		
3–4 per month	26 (7)	19 (19)		
1–2 per week	25 (6)	16 (16)		
3–4 per week	9 (2)	16 (16)		
Almost daily	3 (1)	3 (3)		
Psychotic symptom severity^a^				
Total negative symptoms^e^	12.1 (6.50)	12.6 (5.50)	*t* = −0.76	0.451^b^
Total positive symptoms^d^	12.7 (3.39)	13.4 (3.32)	*t* = −1.77	0.078^b^
Total general symptoms^f^	9.40 (4.37)	9.86 (3.38)	*t* = −1.27	0.261^b^
Total disorganisation symptoms^g^	5.10 (3.15)	5.56 (2.99)	*t* = −1.35	0.180^b^
Antipsychotics users, *n* (%)	78 (20%)	24 (24%)	*χ* ^2^ = 0.4	0.527^c^
Antipsychotic medication dosage (mg/day CPZ)^a^	138 (118)	195 (270)	*t* = 2.16	0.145^b^

*Note*: Psychotic symptom severity was measured with the SOPS. Twenty‐eight CHR‐P did not have a baseline MRI but did have follow‐up scans, which are included in the table. Significant differences are in bold and indicated with an asterisk.

Abbreviations: CHR‐P, clinical high‐risk for psychosis; CPZ, chlorpromazine; MRI, magnetic resonance imaging; SOPS, scale of psychosis‐risk symptoms.

^a^ Values are mean (standard deviation).

^b^ Independent *t*‐test.

^c^ Chi‐squared test.

^d^ Data were missing for one non‐smoking CHR‐P.

^e^ Data were missing for six smoking and three non‐smoking CHR‐P.

^f^ Data were missing for seven smoking and three non‐smoking CHR‐P.

^g^ Data were missing for seven smoking and two non‐smoking CHR‐P.

For within‐network connectivity, we first conducted analyses of covariance (ANCOVA) to test for baseline differences in mean network strength between CHR‐P participants who smoked and those who did not. Mean network strength was the dependent variable, and smoking group was the dichotomous independent variable. Sex, age, cannabis use status, alcohol use status and MRI site were included as covariates. Type III sums of squares and orthogonal contrasts were used. This was repeated for all four networks of interest, with false discovery rate (FDR) correction (Benjamini–Hochberg) for multiple comparisons. For longitudinal analyses, we used linear mixed models (LMMs) to evaluate changes in mean network strength of the DMN, SN, left FPN (LFPN) and right FPN (RFPN) over time between smoking and non‐smoking CHR‐P, using a group × time effect. Linear mixed‐effect models were implemented in RStudio using the lme4 package. Data were included in the models if both MRI and smoking status data were available for at least one time point, as mixed modelling allows valid estimates to be calculated for missing data under the assumption of missing at random. We added random intercepts for subjects and random slopes for time in all models. Covariates of sex, age, cannabis use status and MRI site were added as fixed effects. Age was mean‐centred to improve model performance and interpretability.^54^ A step‐by‐step procedure was employed to incorporate each variable, while the model fit was evaluated using the Akaike information criterion for comparison. Final models were fitted using restricted maximum likelihood estimation. In all mixed‐model analyses, *p* values were calculated using the Satterthwaite method. Residual plots were visually inspected to evaluate deviations from homoscedasticity or normality. This longitudinal analysis was also repeated for each of the four networks. To correct for multiple comparisons, the FDR correction was applied to the four networks of interest across all main analyses.

For between‐network connectivity, ANCOVAs were performed for the six combinations of the networks of interest to test for baseline differences in mean between‐network connectivity strength between smoking and non‐smoking CHR‐P. Analysis was done as described above, with sex, age, cannabis use status, alcohol use status and MRI site included as covariates, but with mean between‐network connectivity as the dependent variable. Next, we used LMMs to evaluate changes in between‐network functional connectivity strength over time between smoking and non‐smoking CHR‐P, using a group × time effect and the covariates age, sex, cannabis use status, alcohol use status and MRI site. To correct for multiple comparisons, the FDR correction was applied to the six combinations of networks of interest across all main analyses.

Missing data on cannabis use were imputed with multiple imputation by chained equations using the mice package^55^ in RStudio. Five datasets with a maximum of 50 iterations for each imputed dataset and the predictive mean matching method were used for imputation, and the seed value from random number generation was set to 500.

### Sensitivity and exploratory analyses

To account for the effects of an unbalanced distribution between CHR‐P smokers and non‐smokers in our main analysis, we conducted a sensitivity analysis using equally distributed samples (*n* = 94 for both groups) matched for age, sex, cannabis use and alcohol use. Further, we performed a sensitivity analysis in which subjects were defined as smokers if they reported smoking more than just occasionally. Although these analyses were not part of the original pre‐registered plan, they were included to strengthen the robustness of our findings. Matching was performed with the MatchIt package^56^ using R.

We exploratively performed the within‐ and between‐network connectivity analyses at baseline as described above for the eight remaining canonical resting‐state networks (see figure S1), correcting for multiple comparisons, to investigate whether effects extended beyond our networks of interest. Lastly, exploratory analyses were performed at baseline to evaluate differences in within‐ and between‐network connectivity between CHR‐P and controls.

## RESULTS

### Demographics and baseline characteristics

**FIGURE 2:**
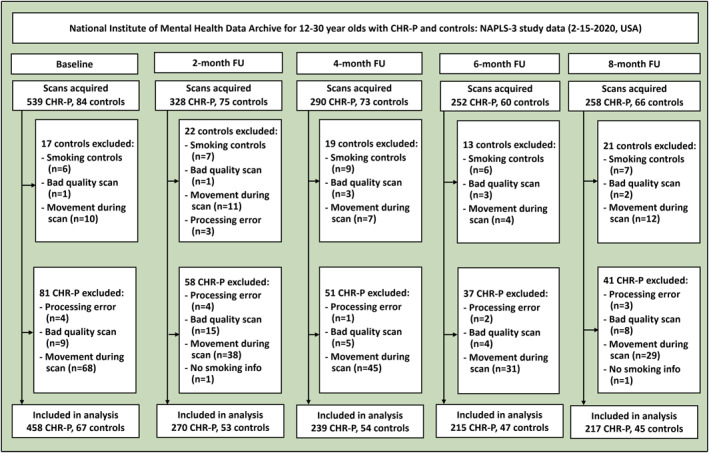
Number of participants and reasons for exclusion for the analysis. The flowchart shows visit‐level counts; participants who missed earlier scans or whose scans failed may appear at later visits, which explains non‐monotonic counts. Smoking controls were excluded at baseline (*n* = 6). If they started smoking at follow‐up (*n* = 1 at month 2 [M2], *n* = 3 at month 4 [M4], *n* = 1 at month 8 [M8]), that scan and all subsequent scans were removed, but prior scans were used. We excluded participants due to having no information on smoking behaviour (*n* = 1 at M2, *n* = 1 at M8), processing errors (*n* = 4 at baseline, *n* = 7 at M2, *n* = 1 at M4, *n* = 2 at month 6 [M6], *n* = 3 at M8), not passing MRI quality assessment (other than movement; *n* = 10 at baseline, *n* = 16 at M2, *n* = 8 at M4, *n* = 7 at M6, *n* = 10 at M8), or moved too much during scanning (*n* = 78 at baseline, *n* = 49 at M2, *n* = 52 at M4, *n* = 35 at M6, *n* = 41 at M8). CHR‐P, clinical high‐risk for psychosis; FU, follow‐up; NAPLS‐3, North American Prodrome Longitudinal Study 3.

In the baseline analysis, 486 CHR‐P individuals were included, comprising 101 smokers and 385 non‐smokers. An additional 71 non‐smoking controls were included. Controls smoking at baseline (*n* = 6), and if they started smoking at follow‐up (*n* = 1 at month 2 [M2], *n* = 3 at month 4 [M4], *n* = 1 at month 8 [M8]), were excluded. Scans performed before the start of smoking were retained. See figure 2 for an exclusion flowchart and further reasons for exclusion. Missing data on cannabis use were imputed for one subject at one time point (non‐smoking CHR‐P, at M2. Data at M4, M6 and M8 were available).

Smoking CHR‐P participants were significantly older, were more often male, had more years of education, used cannabis more frequently and heavily and consumed alcohol more often and in greater quantities (*p* < 0.001). Baseline demographic and clinical characteristics of the CHR‐P participants are summarised in table 1. See table S1 for the baseline demographic and clinical characteristics of the sample, including CHR‐P and controls. In total, 1128 follow‐up scans were performed (table S2). At baseline, 21% of the CHR‐P participants smoked, of which most were occasional smokers (58%; table 1), which stayed relatively consistent over time (table S3). In total, 31 (8%) CHR‐P individuals started smoking, 29 (29%) stopped somewhere during follow‐up and 26 (5%) CHR‐P individuals fluctuated between smoking and non‐smoking.

### Within‐network strength

**FIGURE 3:**
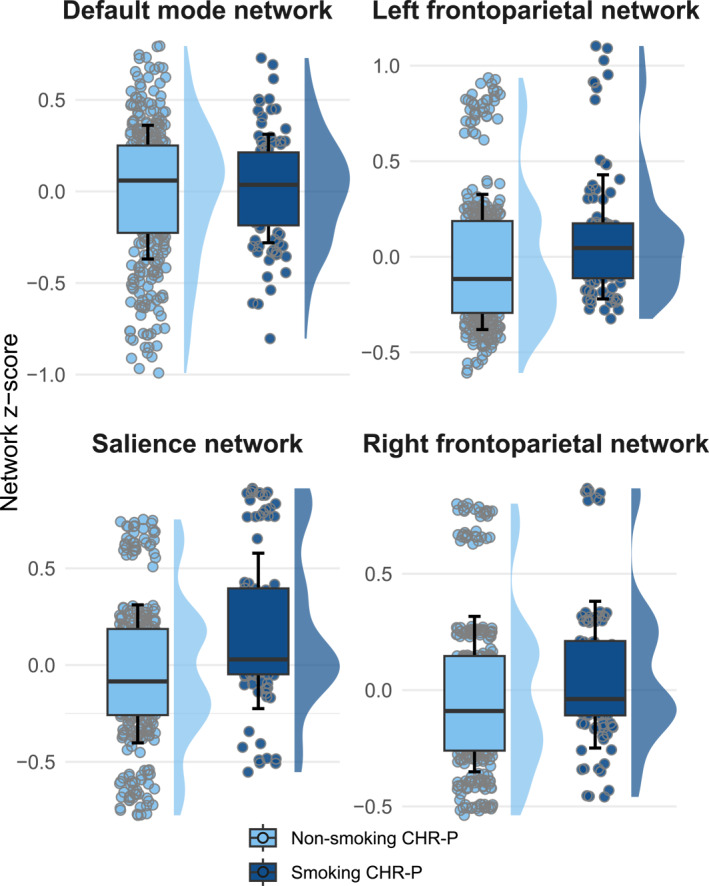
Boxplots with data points and violin plots showing crude *z*‐scores for within‐network strength at baseline for each network of interest (i.e., default mode network, salience network, and left and right frontoparietal network) in smoking and non‐smoking CHR‐P individuals. Boxplots present the group mean ± one standard deviation. Violin plots illustrate the kernel density distribution of the data. CHR‐P, clinical high‐risk for psychosis.

At baseline, smoking CHR‐P had stronger connectivity within the SN than non‐smoking CHR‐P (*F*(1, 444) = 4.175, *p* = 0.041), but this did not survive multiple comparisons correction (*p*_FDR_ = 0.164; figure 3). There were no significant differences at baseline in within‐network strength for any of the other networks of interest (table 2). Longitudinal analyses showed no significant group or time‐by‐group effects on within‐network functional connectivity between CHR‐P smokers and CHR‐P non‐smokers (see figure S2 and table S4).

**TABLE 2:** Differences at baseline in within‐ and between‐network strength of CHR‐P non‐smoking and smoking groups

Network	Non‐smoking CHR‐P (*n* = 364)	Smoking CHR‐P (*n* = 94)	df	*F*	*p*	*p*_FDR_
Within‐network strength, mean *z*‐score (SD)						
DMN	1.410 (0.077)	1.410 (0.074)	1,444	0.034	0.855	0.855
SN	1.200 (0.105)	1.220 (0.083)	**1,444**	**4.175**	0.041*	0.164
LFPN	0.931 (0.073)	0.941 (0.061)	1,444	1.422	0.233	0.466
RFPN	1.130 (0.086)	1.140 (0.077)	1,444	0.573	0.449	0.600
Between‐network strength, mean *z*‐score (SD)						
DMN × LFPN	0.047 (0.256)	−0.002 (0.252)	1,444	2.852	0.092	0.184
DMN × RFPN	0.096 (0.255)	0.043 (0.259)	1,444	3.205	0.074	0.184
DMN × SN	−0.561 (0.299)	−0.549 (0.258)	1,444	0.133	0.715	0.715
LFPN × RFPN	0.459 (0.266)	0.418 (0.285)	1,444	1.866	0.173	0.220
LFPN × SN	−0.117 (0.278)	−0.053 (0.269)	**1,444**	**4.357**	0.037*	0.184
RFPN × SN	0.067 (0.279)	0.112 (0.266)	1,444	1.780	0.183	0.220

*Note*: Results of the ANCOVA analyses. Age, sex, cannabis use (yes/no), alcohol use (yes/no), and MRI site were added as covariates. For statistical analysis, network strength *z*‐scores for the DMN, SN, LFPN, RFPN, and LFPN × RFPN were transformed with a Yeo‐Johnson transformation to correct for negatively skewed non‐normal distribution. Mean *z*‐scores are untransformed and uncorrected for covariates. Significant differences are in bold and indicated with an asterisk.

Abbreviations: ANCOVA, analysis of covariance; CHR‐P, clinical high‐risk for psychosis; df, degrees of freedom; DMN, default mode network; LFPN, left frontoparietal network; RFPN, right frontoparietal network; SD, standard deviation; SN, salience network.

*p < 0.05.

### Between‐network functional connectivity

At baseline, smoking CHR‐P had stronger connectivity between the left and right FPN (*F*(1, 444) = 4.357, *p* = 0.037) compared to non‐smoking CHR‐P, but this did not survive multiple comparisons correction (*p*_FDR_ = 0.184). There were no significant differences in functional network connectivity correlations between smoking and non‐smoking CHR‐P across any other network combinations (table 2; figure S3). Longitudinal analyses showed no significant group or time‐by‐group effects on between‐network functional connectivity between CHR‐P smokers and CHR‐P non‐smokers (table S5).

### Sensitivity and exploratory analyses

Similar to our main analysis, sensitivity analyses in groups matched for age, sex and cannabis use showed no significant differences surviving multiple comparisons in within‐ or between‐network connectivity strength (tables S6 and S7). Analyses in which subjects were defined as smokers if they reported smoking more than just occasionally (*n* = 42) again showed increased SN connectivity in smoking CHR‐P compared to nonsmoking CHR‐P participants at baseline (*F*(1, 444) = 4.391, *p* = 0.037), which also did not remain significant after multiple comparisons correction (*p*_FDR_ = 0.148; table S8). No significant between‐network connectivity differences were observed across any network combinations (table S9).

No significant differences in within‐ or between‐network connectivity strength were observed between smoking and non‐smoking CHR‐P participants for the remaining eight networks after correction for multiple comparisons. Furthermore, there were no significant group differences in within‐ and between‐network connectivity between CHR‐P and non‐smoking controls (tables S10 and S11).

## DISCUSSION

### Main findings

The current study sought to investigate the impact of smoking on resting state functional connectivity between smoking and non‐smoking CHR‐P individuals, as the high prevalence of smoking is associated with worse clinical outcomes in schizophrenia, including cognitive functioning. Smoking behaviour in CHR‐P individuals was mild and not associated with statistically significant differences in within‐ and between‐network functional connectivity of the DMN, SN and FPN in CHR‐P participants, both in cross‐sectional as well as longitudinal analyses.

In the current study, smoking in CHR‐P individuals was not associated with functional connectivity vulnerabilities or changes in these networks. These findings might indicate that mild smoking behaviour does not substantially affect these major functional brain networks prior to the onset of psychosis. Interestingly, given that large‐scale network functional connectivity is associated with cognition,^27^, ^57^ this is consistent with a previous clinical study showing no association between smoking and cognitive functioning in CHR‐P individuals.^58^ Our large sample size and longitudinal within‐subject assessments lend strength and reliability to the results, suggesting that any true effects of smoking on these networks, if any, are likely small in this population.

This contrasts with earlier studies, which reported reduced connectivity within the DMN, FPN and SN in smokers and patients with schizophrenia, and increased connectivity between the DMN and FPN in smoking compared to non‐smoking patients with schizophrenia.^25^, ^33^, ^34^, ^40^ Two factors may account for this discrepancy. First, prior studies involved individuals with psychosis, and smoking‐related network changes may emerge or intensify after psychosis onset. Second, our sample was relatively young and included predominantly light smokers with a relatively short smoking period, in contrast to prior studies that focused on heavier and often older smokers with a longstanding smoking history.^25^, ^34^, ^40^ The lower smoking prevalence, approximately 20% lower than reported in other studies,^20^ may be explained by the relatively young age of our sample and their relatively high functioning status, given the demands of this longitudinal, MRI‐intensive study. The characteristics of our sample suggest a lower cumulative nicotine exposure, which may have been insufficient to be associated with detectable network connectivity changes, as both smoking duration and intensity have been linked to progressive connectivity changes.^25^, ^34^ Thus, our findings suggest that smoking is not strongly linked to within‐ and between‐network connectivity differences in CHR‐P, but the limited nicotine exposure and early illness stage, factors inherent to CHR‐P populations, may have contributed to the absence of significant differences. A structural analysis in the same cohort also showed no significant differences,^59^ further suggesting that smoking may not be associated with neurobiological alterations in this group, or that such effects may require heavier nicotine exposure to be detectable. Future research should extend current findings by including a sample with higher smoking intensity and duration to further investigate the development of network connectivity differences.

Contrary to our hypothesis, we observed higher connectivity within the SN and between the left and right FPN at baseline in CHR‐P smokers, although this did not survive multiple comparisons correction. Sensitivity analyses that included only heavier smokers (i.e., those smoking more than occasionally) yielded similar results (tables S8 and S9). Future research should investigate dose–response relationships and longitudinal changes to determine whether smoking‐related network alterations, particularly within the SN, evolve over time, with cumulative nicotine exposure, or in relation to the progression to psychosis.

Additionally, baseline differences in age, sex, cannabis use and alcohol use between smoking and non‐smoking CHR‐P participants warrant careful consideration. Although sensitivity analyses using groups matched for age, sex and cannabis use yielded comparable results, such analyses are limited by reduced statistical power. Nonetheless, the influence of sample composition may have obscured subtle smoking‐related effects. Overall, our results do not indicate a robust relationship between smoking and network connectivity in young CHR‐P individuals, but further research in more balanced samples is warranted to draw more definitive conclusions.

We observed no significant differences in within‐ or between‐network connectivity between CHR‐P individuals and non‐smoking controls. Although this contrasts with some earlier smaller studies reporting significant differences,^35^, ^36^ it aligns with findings from a recent meta‐analysis.^37^ The low conversion rate to psychosis in our sample (13%) may have limited the detection of group differences, as functional alterations might be more pronounced in individuals who later convert, though further research is needed to confirm this. Of the 51 converters, only 11 individuals smoked, precluding further analysis in this study.

### Limitations

A key strength of the current study is the inclusion of a large cohort of CHR‐P individuals, along with the unique opportunity to examine longitudinal neuroimaging data. The repeated assessments enabled us to model within‐individual variability, yielding more robust and reliable findings. Additionally, the inclusion of data from nine different sites enhances the generalisability of the findings. Nevertheless, in addition to the limitations mentioned above, several others warrant acknowledgement. The study was limited by the lack of detailed information on smoking behaviour, particularly regarding the age of smoking initiation and the timing of the last cigarette prior to the scan. The timing of the last cigarette before the scan is important to account for because both acute nicotine effects and withdrawal have an effect on brain function,^60^ and we were unable to account for or investigate these effects. Possibly, acute effects temporarily mitigate some of the detrimental effects of chronic nicotine exposure on brain networks in patients. Moreover, the absence of data on smoking onset prevented calculation of pack‐years, a key measure of cumulative exposure that could have allowed for a more detailed analysis and helped detect more pronounced differences. We also did not control for antipsychotic medication use, which is known to modulate aberrant functional networks in patients with schizophrenia.^61^, ^62^ However, in our study, few CHR‐P participants were on antipsychotic medication, and when they were, doses were low and comparable between smokers and non‐smokers (table 1). Given the group's young age, prolonged medication use is unlikely. Furthermore, generalisability may be limited, as the sample likely included primarily high‐functioning individuals who were able to provide consent and complete the study. Finally, only six control participants were smokers, which led us to exclude them from group comparisons. Including smoking controls could have facilitated an exploration of the differential association of smoking and network changes between CHR‐P individuals and controls, potentially contributing to a more nuanced understanding of how smoking is associated with potentially different brain connectivity in these groups. This is especially of interest, as recent research highlighted that smoking may be differentially associated with neural dynamics^39^ and neurometabolite levels^63^ in individuals with and without schizophrenia, highlighting the complex neurobiological mechanisms underlying this interaction.

### Implications

In conclusion, our findings do not support that mild smoking is associated with baseline or eight‐month follow‐up within‐ and between‐network functional connectivity of the DMN, SN and FPN in CHR‐P individuals. This suggests that smoking is not associated with large‐scale network changes at this early stage of illness. However, longitudinal studies with higher smoking intensity and longer follow‐up than eight months are needed to clarify whether network alterations emerge with heavier and more longstanding use or illness progression. Furthermore, to fully understand the co‐occurrence of smoking and psychosis, it is important to explore alternative neurobiological pathways. Diffusion‐weighted MRI, for example, may offer valuable insights, as smoking is associated with exacerbating white matter integrity reduction in individuals with schizophrenia.^64^, ^65^ Moreover, integrating micro‐scale molecular information with macro‐scale neuroimaging may further elucidate the hierarchical organisation of the brain.^66^ In this context, nAChR imaging is especially relevant due to its role in schizophrenia and smoking behaviour. Patients with psychosis share common genetic variants with tobacco addiction in the cholinergic receptor nicotinic alpha gene cluster, which encodes nAChRs,^67^, ^68^ and also tend to exhibit reduced receptor expression,^69^ which may underlie their elevated smoking rates. Combining nAChR imaging with MRI enables investigation of the role of nAChR in smoking in combination with psychosis (risk) and the potential influence on brain changes at the macro scale, thereby enhancing our understanding of the neurobiological mechanisms underlying the co‐occurrence.

## AUTHOR CONTRIBUTIONS

The study was designed and supervised by Elsmarieke van de Giessen, Lieuwe de Haan, Marieke van der Pluijm, Tim Ziermans and Jentien Vermeulen. Jentien Vermeulen acted as the guarantor. Data analyses and interpretation were performed by Merel Koster and Romy Veelers under the supervision of Marieke van der Pluijm, Guido van Wingen, and Jentien Vermeulen. Merel Koster wrote the original draft and revised it critically for intellectual content by all authors. All authors have read and agreed to the published version of the manuscript. Tim Ziermans and Jentien Vermeulen are the joint last authors.

## CONFLICT OF INTEREST STATEMENT

The authors declare no conflicts of interest.

## ETHICS STATEMENT

The study procedures were approved by institutional review boards at all participating sites. The current study involved only a secondary analysis of de‐identified publicly available data (through the NDA). Therefore, no separate ethical approval was required for the current study.

## PATIENT CONSENT FOR PUBLICATION

Not applicable.

## Supporting information

Supporting Information S1

## Data Availability

Data used in this study are publicly available through the National Institute of Mental Health (NIMH) Data Archive (NDA). The NDA Study DOI for this study is 10.15154/j0hz‐6a55.
