# Correction to “Resting‐state connectivity and tobacco smoking in clinical high‐risk for psychosis (NAPLS‐3)”

**DOI:** 10.1002/gps3.70040

**Published:** 2026-06-21

Koster M, van der Pluijm M, Veelers R, van de Giessen E, de Haan L, van Wingen G, et al. Resting‐state connectivity and tobacco smoking in clinical high‐risk for psychosis (NAPLS‐3). *Gen Psychiatr*. 2026;39:e70002. https://doi.org/10.1002/gps3.70002

1. In “Methods” of the “Abstract” section, the text “486 CHR‐P non‐smokers and 101 CHR‐P smokers were included” was incorrect.

This should have read: “364 CHR‐P non‐smokers and 94 CHR‐P smokers were included”.

2. In the first paragraph of “Demographics and baseline characteristics” of the “Results” section, the text “In the baseline analysis, 486 CHR‐P individuals were included, comprising 101 smokers and 385 non‐smokers. An additional 71 non‐smoking controls were included” was incorrect.

This should have read: “In total, 486 CHR‐P individuals were included, comprising 101 smokers and 385 non‐smokers. An additional 71 non‐smoking controls were included. Not all subjects had a baseline MRI, and the baseline analysis included 364 CHR‐P non‐smokers, 94 CHR‐P smokers, and 67 controls”.

3. In paragraph 2 of “Demographics and baseline characteristics” of the “Results” section, the text “Baseline demographic and clinical characteristics of the CHR‐P participants are summarised in table 1” was incorrect.

This should have read: “Demographic and clinical characteristics of the CHR‐P participants are summarised in table 1”.

4. In paragraph 2 of “Demographics and baseline characteristics” of the “Results” section, the text “See table S1 for the baseline demographic and clinical characteristics of the sample” was incorrect.

This should have read “See table S1 for the demographic and clinical characteristics of the sample”.

5. In paragraph 2 of “Demographics and baseline characteristics” of the “Results” section, the text “At baseline, 21% of the CHR‐P participants smoked” was incorrect.

This should have read: “21% of the CHR‐P participants smoked”.

We apologize for this error.

