# Supplementary material for: Resting‐state connectivity and tobacco smoking in clinical high‐risk for psychosis (NAPLS‐3)
Source: Gen Psychiatr. 2026 Mar 11;39(1):e70002. doi: 10.1002/gps3.70002 (PMC13015833; doi:10.1002/gps3.70002)
Supplement: Supplementary file 1 — Supporting Information S1 [file GPS3-39-e70002-s001.docx]

**Supplement to: Resting-state connectivity and tobacco smoking in clinical high-risk for psychosis (NAPLS 3)**

**Figure S1** Twelve canonical resting-state networks, of which the default mode network, salience network, left frontoparietal network and right frontoparietal network were selected for the group-level analyses.


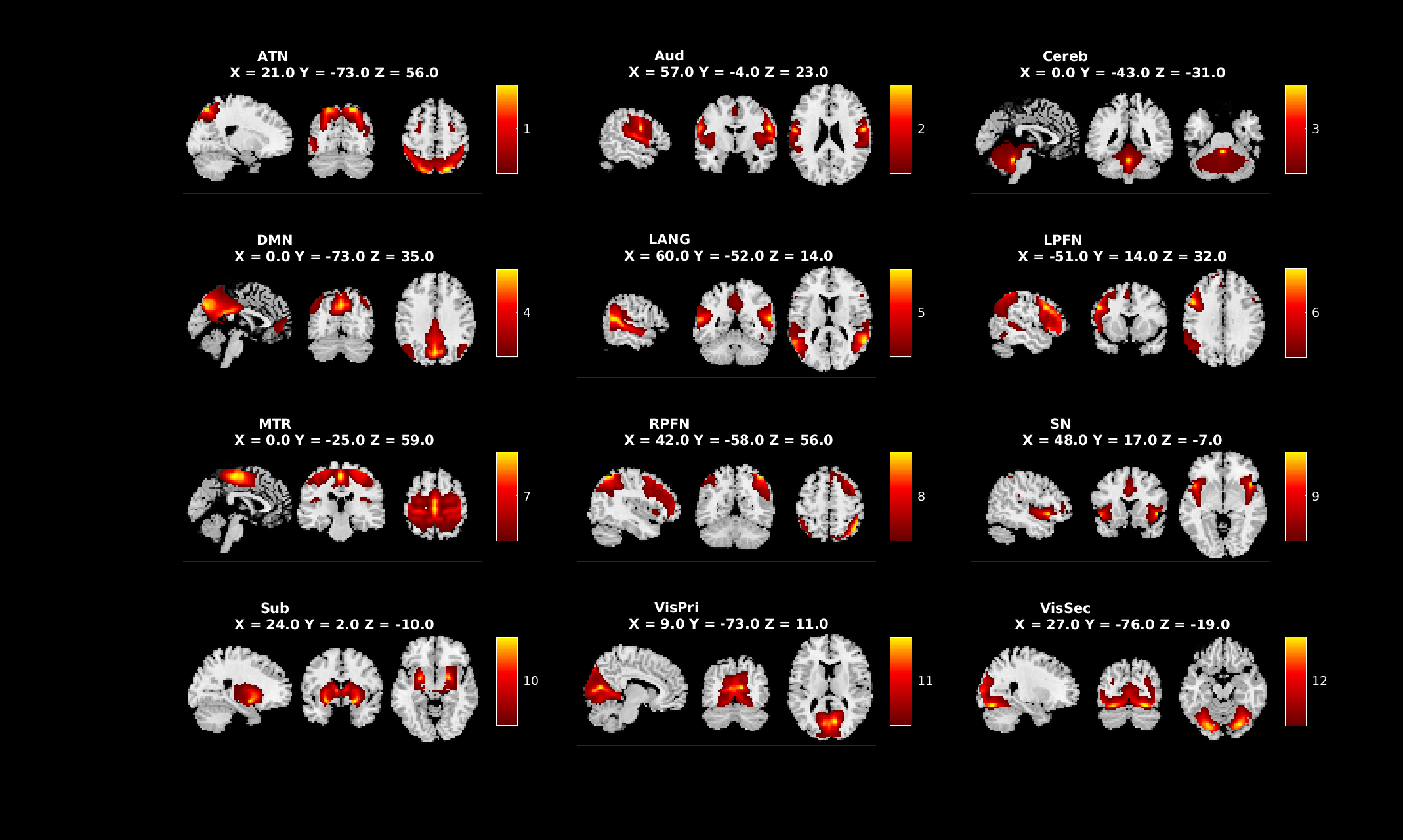


Abbreviations: ATN, attention network; Aud, auditory network; Cereb, cerebral network; DMN, default mode network; LANG, language network; LPFN, left frontoparietal network; MTR, motor network; RPFN, right frontoparietal network; SN, salience network; Sub, subcortical network; VisPri, primary visual network; VisSec, secondary visual network.

**Table S1** Demographical and clinical characteristics of CHR-P participants and controls at baseline.

|  | **CHR-P (n=486)** | **Controls (n=71)** | **Statistic** | **p** |
| --- | --- | --- | --- | --- |
| Age in years^1^ | 18.6 (3.95) | 18.7 (3.95) | 0.229 | 0.819^2^ |
| Sex (N female, %) | 216 (44%) | 38 (54%) | 1.71 | 0.191^3^ |
| Years of education^1^ | 11.8 (3.04)^4^ | 12.5 (3.30) | 1.71 | 0.091^3^ |
| Smoking frequency (N, %) |  |  | **18.0** | **0.001*^3^** |
| No use | 385 (79%) | 71 (100%) |  |  |
| Occasionally | 59 (12%) | 0 (0%) |  |  |
| <10 cigarettes per day | 22 (5%) | 0 (0%) |  |  |
| 10-25 cigarettes per day | 18 (4%) | 0 (0%) |  |  |
| >25 cigarettes per day | 2 (0%) | 0 (0%) |  |  |
| Cannabis users (N, %) | 128 (26%) | 6 (8%) | **9.89** | **0.002*^3^** |
| Cannabis use severity (N, %) |  |  | **12.5** | **0.029*^3^** |
| No use | 358 (74%) | 65 (92%) |  |  |
| Once/twice per month | 58 (12%) | 4 (6%) |  |  |
| 3-4 times per month | 16 (3%) | 0 (0%) |  |  |
| 1-2 times per week | 22 (5%) | 0 (0%) |  |  |
| 3-4 times per week | 19 (4%) | 2 (3%) |  |  |
| Almost daily | 13 (3%) | 0 (0%) |  |  |
| Alcohol users (N, %) | 194 (40%) | 33 (46%) | 0.849 | 0.357^3^ |
| Alcohol use severity (N, %) |  |  | 6.11 | 0.296^3^ |
| No use | 292 (60%) | 38 (54%) |  |  |
| Once/twice per month | 77 (16%) | 16 (23%) |  |  |
| 3-4 times per month | 45 (9%) | 7 (10%) |  |  |
| 1-2 times per week | 41 (8%) | 9 (13%) |  |  |
| 3-4 times per week | 25 (5%) | 1 (1%) |  |  |
| Almost daily | 6 (1%) | 0 (0%) |  |  |
| Psychotic symptom severity^1^ |  |  |  |  |
| Total negative symptoms | 12.2 (6.31)^5^ | 1.30 (2.10) | **-28.7** | **<0.001*^2^** |
| Total positive symptoms | 12.9 (3.38)^4^ | 0.83 (1.29) | **-55.6** | **<0.001*^2^** |
| Total general symptoms | 9.49 (4.18)^6^ | 1.25 (2.05) | **-26.6** | **<0.001*^2^** |
| Total disorganization symptoms | 5.19 (3.12)^5^ | 0.51 (0.88) | **-26.5** | **<0.001*^2^** |
| Antipsychotic users (N, %) | 102 (21%) | 0 (0%) | **16.9** | **<0.001*^3^** |
| Antipsychotic medication dosage (mg/day CPZ)^1^ | 152 (167) | 0 (0) | **58.9** | **<0.001*^2^** |

All controls were non-smokers. 28 CHR-P and 4 controls did not have a baseline MRI, but did have follow-up scans, and are included in the table. Abbreviations: CHR-P, clinical high-risk for psychosis; CPZ, chlorpromazine. * p<0.05.

^1^ Values are mean (standard deviation)

^2^ Independent t-test. Corresponding statistic is a t-value.

^3^ Chi-squared test. Corresponding statistic is a X-squared value.

^4^ Data were missing for 1 person

^5^ Data were missing for 9 people

^6^ Data were missing for 10 people

**Table S2.** Number of baseline and follow-up measurements per group

|  | **Baseline** | **2 month FU** | **4 month FU** | **6 month FU** | **8 month FU** |
| --- | --- | --- | --- | --- | --- |
| **Controls** | 67 (13%) | 53 (16%) | 54 (19%) | 47 (18%) | 45 (17%) |
| **Non-smoking CHR-P** | 364 (69%) | 219 (68%) | 187 (67%) | 175 (67%) | 170 (65%) |
| **Smoking CHR-P** | 94 (18%) | 51 (16%) | 40 (14%) | 40 (15%) | 47 (18%) |
| **Total** | 525 | 323 | 281 | 262 | 262 |

Smokers were defined as those who smoke at least occasionally (≥ 1 on the tobacco severity scale), and non-smokers as participants who do not smoke at all (0 on the tobacco severity scale). Participants were classified as smokers from the moment they start smoking. Abbreviations: CHR-P, clinical high-risk for psychosis participants; FU, follow-up.

**Table S3.** CHR-P participants categorized per smoking behavior across the baseline and follow-up measurements.

|  | **Baseline** | **2 months** | **4 months** | **6 months** | **8 months** |
| --- | --- | --- | --- | --- | --- |
| **Tobacco addiction scale (n, %)** |  |  |  |  |  |
| **1** | 364 (79%) | 219 (81%) | 187 (82%) | 175 (81%) | 170 (78%) |
| **2** | 83 (18%) | 46 (17%) | 38 (17%) | 40 (19%) | 44 (20%) |
| **3** | 3 (1%) | 2 (1%) | 0 (0%) | 0 (0%) | 2 (1%) |
| **4** | 8 (2%) | 3 (1%) | 2 (1%) | 0 (0%) | 1 (0%) |
| **Tobacco severity scale (n, %)** |  |  |  |  |  |
| **0** | 364 (79%) | 219 (81%) | 187 (82%) | 175 (81%) | 170 (78%) |
| **1** | 53 (12%) | 32 (12%) | 23 (10%) | 22 (10%) | 24 (11%) |
| **2** | 21 (5%) | 10 (4%) | 11 (5%) | 11 (5%) | 12 (6%) |
| **3** | 18 (4%) | 7 (3%) | 5 (2%) | 7 (3%) | 10 (5%) |
| **4** | 2 (0%) | 2 (1%) | 1 (0%) | 0 (0%) | 1 (0%) |

Tobacco addiction scale, assessed by clinician: 1= abstinent, 2= use without impairment, 3= abuse, 4= dependence; Tobacco smoking severity scale, through self-report: 0=no use, 1= occasionally, 2= less than 10 cigarettes per day, 3= Between 10 and 25 cigarettes per day, 4= more than 25 cigarettes per day. Abbreviations: CHR-P, clinical high-risk for psychosis participants.

**Figure S2.** Crude within-network strength z-scores at baseline for each network of interest, i.e., default mode network, salience network, and left and right frontoparietal network are shown averaged across subjects. Error bars indicate standard errors. Abbreviations: CHR-P, clinical high-risk for psychosis.


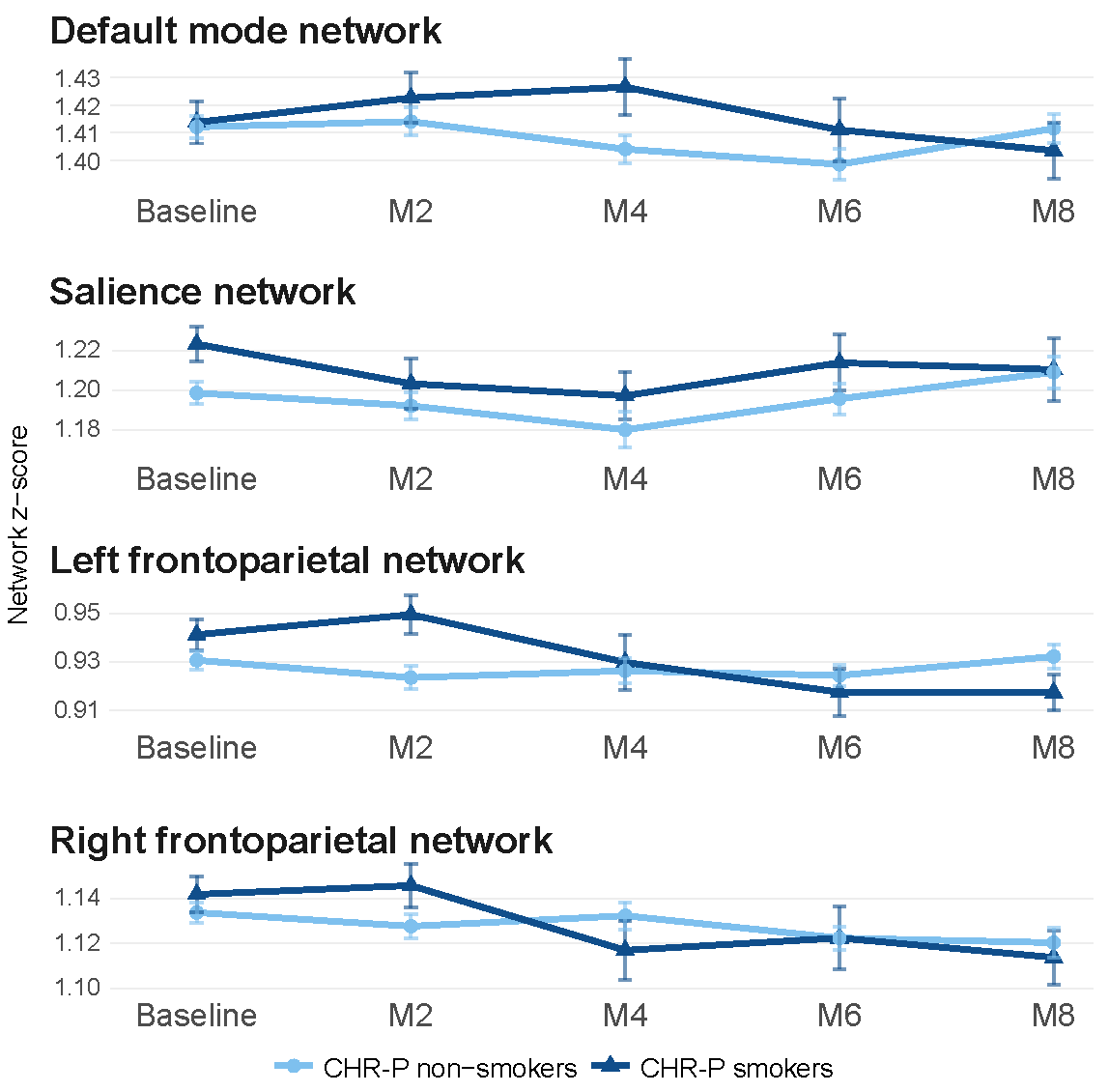


**Table S4.** Longitudinal analysis between CHR-P smokers and CHR-P non-smokers on the effect of smoking over time on within network connectivity.

| **DMN** | **Estimate** | **SE** | **Df** | **t** | **P** | **pFDR** |
| --- | --- | --- | --- | --- | --- | --- |
| Intercept | **-0.311** | **0.115** | **597** | **-2.716** | **0.007*** | **-** |
| Group | 0.037 | 0.130 | 782 | 0.155 | 0.877 | 0.877 |
| Time | -0.023 | 0.017 | 357 | -1.342 | 0.180 | 0.245 |
| Group x Time | 0.029 | 0.038 | 544 | 0.748 | 0.455 | 0.898 |
| **SN** |  |  |  |  |  |  |
| Intercept | -0.162 | 0.114 | 601 | -1.428 | 0.154 | - |
| Group | 0.133 | 0.126 | 764 | 1.062 | 0.289 | 0.578 |
| Time | 0.029 | 0.017 | 345 | 1.332 | 0.184 | 0.245 |
| Group x Time | -0.0002 | 0.040 | 545 | -0.006 | 0.995 | 0.995 |
| **LFPN** |  |  |  |  |  |  |
| Intercept | **-0.318** | **0.116** | **629** | **-2.746** | **0.006*** | **-** |
| Group | *0.234* | *0.127* | *809* | *1.829* | *0.068* | *0.272* |
| Time | -0.013 | 0.016 | 377 | -0.766 | 0.444 | 0.444 |
| Group x Time | *-0.061* | *0.037* | *567* | *-1.658* | *0.097* | 0.388 |
| **RPFN** |  |  |  |  |  |  |
| Intercept | **-0.361** | **0.114** | **631** | **-3.183** | **0.001*** | **-** |
| Group | 0.060 | 0.131 | 811 | 0.456 | 0.648 | 0.864 |
| Time | **-0.040** | **0.017** | **382** | **-2.321** | **0.021*** | *0.084* |
| Group x Time | -0.016 | 0.038 | 580 | -0.421 | 0.674 | 0.899 |

Fixed effects in the models were smoking status*time, sex, age, cannabis use, alcohol use, and MRI site. Random effects were intercepts for subjects and random slopes for time. Significant p-values are in bold. Abbreviations: CHR-P, clinical high-risk for psychosis; df, degrees of freedom; SE, standard error. *p<0.05

**Figure S3.** Difference in baseline functional network connectivity between smoking and non-smoking CHR-P individuals. Positive z-scores correspond to increase in between-network connectivity in smoking CHR-P versus nonsmoking CHR-P. None of the combinations of networks differed significantly (p>0.05, ANCOVA).


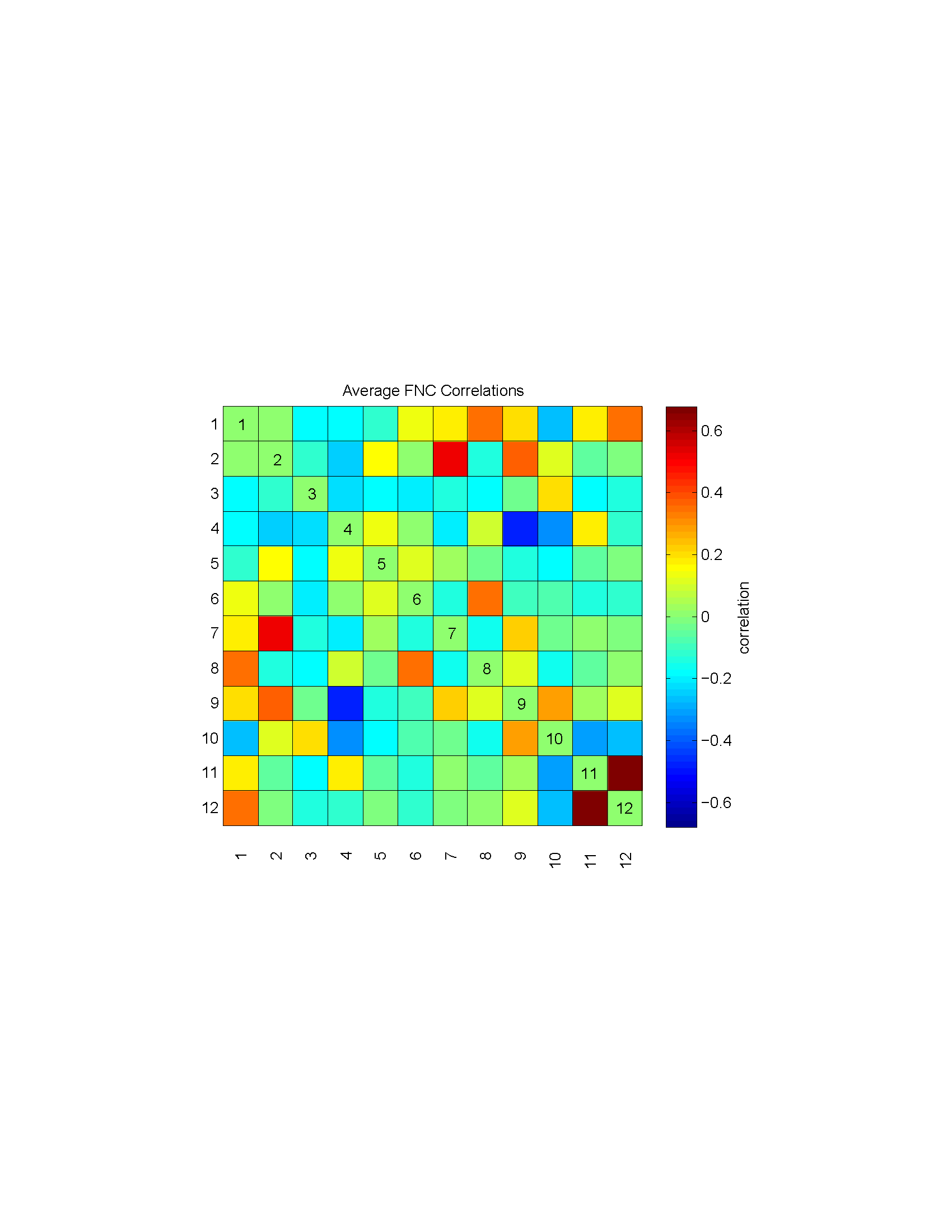


**Table S5.** Longitudinal analyses between CHR-P smokers and CHR-P non-smokers on the effect of smoking over time on between network connectivity.

| **DMN x LPFN** | **Estimate** | **SE** | **Df** | **t** | **P** | **pFDR** |
| --- | --- | --- | --- | --- | --- | --- |
| Intercept | 0.014 | 0.028 | 616 | 0.490 | 0.624 |  |
| Group | -0.021 | 0.035 | 737 | -0.591 | 0.555 | 0.857 |
| Time | -0.001 | 0.005 | 370 | -0.205 | 0.838 | 0.838 |
| Group x Time | -0.003 | 0.013 | 585 | -0.222 | 0.824 | 0.959 |
| **DMN x RPFN** |  |  |  |  |  |  |
| Intercept | **0.092** | **0.029** | **661** | **3.222** | **0.001*** |  |
| Group | -0.040 | 0.035 | 1055 | -1.129 | 0.259 | 0.777 |
| Time | -0.003 | 0.005 | 1057 | -0.711 | 0.477 | 0.715 |
| Group x Time | 0.014 | 0.011 | 1169 | 1.267 | 0.205 | 0.615 |
| **DMN x SN** |  |  |  |  |  |  |
| Intercept | **-0.561** | **0.033** | **631** | **-17.104** | **<0.001*** |  |
| Group | -0.017 | 0.040 | 748 | -0.425 | 0.641 | 0.857 |
| Time | 0.008 | 0.006 | 381 | 1.449 | 0.148 | 0.444 |
| Group x Time | 0.010 | 0.013 | 582 | 0.795 | 0.427 | 0.854 |
| **LPFN x RPFN** |  |  |  |  |  |  |
| Intercept | **0.284** | **0.109** | **633** | **2.596** | **0.010*** |  |
| Group | -0.189 | 0.391 | 773 | -1.355 | 0.176 | 0.777 |
| Time | 0.018 | 0.019 | 373 | 0.961 | 0.337 | 0.674 |
| Group x Time | 0.071 | 0.042 | 575 | 1.670 | 0.096 | 0.576 |
| **LPFN x SN** |  |  |  |  |  |  |
| Intercept | -0.012 | 0.123 | 629 | -1.057 | 0.291 |  |
| Group | 0.049 | 0.134 | 779 | 0.366 | 0.714 | 0.857 |
| Time | 0.006 | 0.019 | 377 | 0.328 | 0.743 | 0.838 |
| Group x Time | 0.002 | 0.041 | 590 | 0.052 | 0.959 | 0.959 |
| **RPFN x SN** |  |  |  |  |  |  |
| Intercept | 0.008 | 0.031 | 647 | 0.241 | 0.810 |  |
| Group | 0.004 | 0.038 | 807 | 0.115 | 0.909 | 0.909 |
| Time | **-0.012** | **0.005** | **379** | **-2.292** | **0.022*** | 0.132 |
| Group x Time | -0.006 | 0.013 | 594 | -0.505 | 0.613 | 0.920 |

Fixed effects in the models were smoking status*time, sex, age, cannabis use, alcohol use (yes/no), and MRI site. Random effects were intercepts for subjects and random slopes for time. Significant p-values are in bold.

Abbreviations: CHR-P, clinical high-risk for psychosis; df, degrees of freedom; SE, standard error.* p <0.05

**Table S6.** Differences at baseline in within-network strength in matched groups

|  | **Within-network strength**  **[mean z-score (SD)]** | | **ANCOVA** | | |  |
| --- | --- | --- | --- | --- | --- | --- |
| **Network** | Non-smoking CHR-P (n=94) | Smoking CHR-P (n=94) | *df* | *F* | *p* | *pFDR* |
| **DMN** | 1.41 (0.085) | 1.41 (0.074) | 1,174 | 0.017 | 0.897 | 0.897 |
| **SN** | 1.20 (0.107) | 1.22 (0.083) | **1,174** | **2.34** | **0.128** | **0.512** |
| **LFPN** | 0.935 (0.072) | 0.941 (0.062) | 1,174 | 0.346 | 0.557 | 0.743 |
| **RFPN** | 1.13 (0.088) | 1.14 (0.077) | 1,174 | 0.722 | 0.397 | 0.743 |

Age, sex, cannabis use (yes/no), alcohol use (yes/no), and MRI site were added as covariates. For statistical analysis, network strength z-scores were transformed with a Yeo-Johnson transformation to correct for negatively skewed non-normal distribution. Mean z-scores are untransformed and uncorrected for covariates. Abbreviations: ANCOVA, analysis of covariance; CHR-P, clinical high-risk for psychosis; df, degrees of freedom; DMN, default mode network; LFPN, left frontoparietal network; RPFN, right frontoparietal network; SD, standard deviation; SN, salience network. * p<0.05.

T**able S7.** Differences at baseline in between-network strength in matched groups

|  | **Between-network strength**  **[mean z-score (SD)]** | | **ANCOVA** | | |  |
| --- | --- | --- | --- | --- | --- | --- |
| **Network** | Non-smoking CHR-P (n=94) | Smoking CHR-P (n=94) | *df* | *F* | *p* | *pFDR* |
| **DMN x LPFN** | 0.084 (0.267) | -0.002 (0.252) | **1,174** | **5.047** | **0.026*** | 0.117 |
| **DMN x RPFN** | 0.089 (0.278) | 0.043 (0.259) | 1,174 | 1.327 | 0.251 | 0.502 |
| **DMN x SN** | -0.539 (0.313) | -0.549 (0.258) | 1,174 | 0.067 | 0.796 | 0.832 |
| **LPFN x RPFN** | 0.450 (0.269) | 0.418 (0.285) | 1,174 | 0.754 | 0.386 | 0.579 |
| **LPFN x SN** | -0.134 (0.312) | -0.053 (0.269) | **1,174** | **4.337** | **0.039*** | 0.117 |
| **RPFN x SN** | 0.103 (0.302) | 0.112 (0.266) | 1,174 | 0.045 | 0.832 | 0.832 |

Age, sex, cannabis use (yes/no), alcohol use (yes/no), and MRI site were added as covariates. For statistical analysis, network strength z-scores of LPFNxRPFN and LPFNxSN were transformed with a Yeo-Johnson transformation to correct for negatively skewed non-normal distribution. Mean z-scores are untransformed and uncorrected for covariates.: ANCOVA, analysis of covariance; CHR-P, clinical high-risk for psychosis; df, degrees of freedom; DMN, default mode network; LFPN, left frontoparietal network; RPFN, right frontoparietal network; SD, standard deviation; SN, salience network.

**Table S18.** Differences at baseline in within-network strength for sensitivity analysis in heavier smokers

|  | **Within-network strength**  **[mean z-score (SD)]** | | **ANCOVA** | | | |
| --- | --- | --- | --- | --- | --- | --- |
| **Network** | Non-smoking CHR-P (n=417) | Smoking CHR-P (n=42) | *df* | *F* | *p* | *P_FDR_* |
| **DMN** | 1.41 (0.077) | 1.42 (0.074) | 1,444 | 1.451 | 0.229 | 0.305 |
| **SN** | 1.20 (0.103) | 1.23 (0.085) | **1,444** | **4.391** | **0.037*** | 0.148 |
| **LFPN** | 0.932 (0.072) | 0.946 (0.061) | 1,444 | 1.465 | 0.227 | 0.305 |
| **RFPN** | 1.14 (0.086) | 1.14 (0.075) | 1,444 | 0.000 | 0.985 | 0.985 |

Age, sex, cannabis use (yes/no), alcohol use (yes/no), and MRI site were added as covariates. Subjects were defined as smokers if they reported smoking more than just occasionally. For statistical analysis, network strength z-scores were transformed with a Yeo-Johnson transformation to correct for negatively skewed non-normal distribution Mean z-scores are untransformed and uncorrected for covariates. Abbreviations: ANCOVA, analysis of covariance; CHR-P, clinical high-risk for psychosis; df, degrees of freedom; DMN, default mode network; LFPN, left frontoparietal network; RPFN, right frontoparietal network; SD, standard deviation; SN, salience network. * p<0.05.

T**able S9.** Differences at baseline in between-network strength for sensitivity analysis in heavier smokers

|  | **Between-network strength**  **[mean z-score (SD)]** | | **ANCOVA** | | | |
| --- | --- | --- | --- | --- | --- | --- |
| **Network** | Non-smoking CHR-P (n=417) | Smoking CHR-P (n=42) | *df* | *F* | *p* | *P_FDR_* |
| **DMN x LPFN** | 0.040 (0.256) | 0.005 (0.253) | 1,444 | 0.741 | 0.390 | 0.780 |
| **DMN x RPFN** | 0.086 (0.254) | 0.075 (0.289) | 1,444 | 0.073 | 0.788 | 0.936 |
| **DMN x SN** | -0.559 (0.296) | -0.555 (0.234) | 1,444 | 0.006 | 0.936 | 0.936 |
| **LPFN x RPFN** | 0.447 (0.267) | 0.486 (0.300) | 1,444 | 0.790 | 0.375 | 0.780 |
| **LPFN x SN** | -0.111 (0.282) | -0.040 (0.211) | 1,444 | 2.584 | 0.109 | 0.654 |
| **RPFN x SN** | 0.078 (0.283) | 0.088 (0.205) | 1,444 | 0.055 | 0.814 | 0.936 |

Age, sex, cannabis use (yes/no), alcohol use (yes/no), and MRI site were added as covariates. Subjects were defined as smokers if they reported smoking more than just occasionally. For statistical analysis, network strength z-scores of LPFNxRPFN and LPFNxSN were transformed with a Yeo-Johnson transformation to correct for negatively skewed non-normal distribution Mean z-scores are untransformed and uncorrected for covariates. Abbreviations: ANCOVA, analysis of covariance; CHR-P, clinical high-risk for psychosis; df, degrees of freedom; DMN, default mode network; LFPN, left frontoparietal network; RPFN, right frontoparietal network; SD, standard deviation; SN, salience network.

**Table S10.** Differences at baseline in within-network strength in CHR-P vs controls

|  | **Within-network strength**  **[mean z-score (SD)]** | | **ANCOVA** | | |  |
| --- | --- | --- | --- | --- | --- | --- |
| **Network** | CHR-P (n=458) | Controls (n=67) | *df* | *F* | *p* | *pFDR* |
| **DMN** | 1.45 (0.087) | 1.46 (0.063) | 1,511 | 0.268 | 0.605 | 0.901 |
| **SN** | 1.28 (0.115) | 1.28 (0.099) | 1,511 | 0.015 | 0.901 | 0.901 |
| **LFPN** | 1.10 (0.083) | 1.11 (0.076) | 1,511 | 0.363 | 0.547 | 0.901 |
| **RFPN** | 1.27 (0.096) | 1.28 (0.079) | 1,511 | 0.054 | 0.817 | 0.901 |

Age, sex, cannabis use (yes/no), alcohol use (yes/no), and MRI site were added as covariates. For statistical analysis, network strength z-scores were transformed with a Yeo-Johnson transformation to correct for negatively skewed non-normal distribution. Mean z-scores are untransformed and uncorrected for covariates. Abbreviations: ANCOVA, analysis of covariance; CHR-P, clinical high-risk for psychosis; df, degrees of freedom; DMN, default mode network; LFPN, left frontoparietal network; RPFN, right frontoparietal network; SD, standard deviation; SN, salience network.

T**able S11.** Differences at baseline in between-network strength in CHR-P vs controls

|  | **Between-network strength**  **[mean z-score (SD)]** | | **ANCOVA** | | |  |
| --- | --- | --- | --- | --- | --- | --- |
| **Network** | CHR-P (n=458) | Controls (n=67) | *df* | *F* | *p* | *pFDR* |
| **DMN x LPFN** | 0.008 (0.252) | -0.016 (0.277) | 1,511 | 0.542 | 0.462 | 0.511 |
| **DMN x RPFN** | 0.095 (0.255) | 0.117 (0.214) | 1,511 | 0.476 | 0.491 | 0.511 |
| **DMN x SN** | -0.525 (0.293) | -0.477 (0.316) | 1,511 | 1.594 | 0.207 | 0.511 |
| **LPFN x RPFN** | 0.356 (0.266) | 0.378 (0.271) | 1,511 | 0.432 | 0.511 | 0.511 |
| **LPFN x SN** | -0.088 (0.273) | -0.026 (0.270) | *1,511* | *3.160* | *0.076* | *0.456* |
| **RPFN x SN** | 0.111 (0.274) | 0.085 (0.274) | 1,511 | 0.559 | 0.455 | 0.511 |

Age, sex, cannabis use (yes/no), alcohol use (yes/no), and MRI site were added as covariates. For statistical analysis, network strength z-scores of LPFNxRPFN and LPFNxSN were transformed with a Yeo-Johnson transformation to correct for negatively skewed non-normal distribution. Mean z-scores are untransformed and uncorrected for covariates.: ANCOVA, analysis of covariance; CHR-P, clinical high-risk for psychosis; df, degrees of freedom; DMN, default mode network; LFPN, left frontoparietal network; RPFN, right frontoparietal network; SD, standard deviation; SN, salience network.
